# The taxonomy of an Australian nodavirus isolated from mosquitoes

**DOI:** 10.1371/journal.pone.0210029

**Published:** 2018-12-31

**Authors:** David Warrilow, Bixing Huang, Natalee D. Newton, Jessica J. Harrison, Karyn N. Johnson, Weng Kong Chow, Roy A. Hall, Jody Hobson-Peters

**Affiliations:** 1 Public Health Virology Laboratory, Queensland Health Forensic and Scientific Services, Archerfield, Queensland; 2 Australian Infectious Diseases Research Centre, School of Chemistry and Molecular Biosciences, The University of Queensland, St Lucia, Queensland, Australia; 3 School of Biological Sciences, The University of Queensland, St Lucia, Queensland, Australia; 4 Australian Defence Force Malaria and Infectious Disease Institute, Queensland, Australia; Keele University Faculty of Natural Sciences, UNITED KINGDOM

## Abstract

We describe a virus isolated from *Culex annulirostris* mosquitoes in Australia. Phylogenetic analysis of its RNA-dependent RNA polymerase sequence and that of other related viruses revealed 6 clades, two of which corresponded wholly or partly with existing genera in the family *Nodaviridae*. There was greater genetic diversity within the family than previously recognized prompting us to suggest that additional genera should be considered within the family.

## Introduction

Nodaviruses are positive-sense RNA viruses with bipartite genomes which are capped but not polyadenylated [1]. There are currently two genera recognized: *Alphanodavirus* (5 species) and *Betanodavirus* (4 species). The alphanodaviruses primarily infect insects, and betanodaviruses infect fish. Historically, the earliest isolations were of the alphanodaviruses in Australasia, initially with *Nodamura virus* (NoV) in Japan [2]; *Flock House virus* (FHV), and then *Black beetle virus* (BBV), and *Boolarra virus* (BoV) [3]. More recently, *Pariacoto virus* (PaV) [4] was isolated from a sample from Peru; the first alphanodavirus identified outside Australasia. The four recognized species in the betanodaviruses are *Barfin flounder nervous necrosis virus* (BFNNV), *Redspotted grouper nervous necrosis virus* (RGNNV), *Striped jack nervous necrosis virus* (SJNNV), and *Tiger puffer nervous necrosis virus* (TPNNV) [3]. In 2014, a nodavirus named mosinovirus (MoNV) was isolated from mosquito species of the *Culex* genus [5]. Currently, MoNV is not yet recognized as a species and has not been assigned to a genus. Most recently, a large number of nodavirus-like sequences were identified in a large-scale sequencing study of invertebrates [6]. This study has greatly enlarged our knowledge of the genetic diversity of this group of viruses, and will be complemented by future research on their biology.

The two genomic RNAs of nodaviruses, a ~3.1 kb RNA1 and ~1.4 kb RNA2 respectively, encode for two open reading frames (ORFs) [1]. RNA1 ORF1 (protein A) encodes the RNA-dependent RNA polymerase (RdRp) and the RNA2 ORF1 encodes the capsid protein. A third RNA identified in infected cells is sub-genomic and derived from RNA1, and potentially encodes two small proteins: B1 and B2. B2 has been linked to suppression of virus-inhibitory RNAi activity [7]. Interestingly, a fourth RNA of MoNV is also sub-genomic and encodes B2-like RNAi suppressor activity [5]. Also of interest is the observation that the MoNV RNA2 capsid seems to have originated from a distantly-related virus family as indicated by the genetic distance separating this genome segment from other nodaviruses [5].

In this work we describe a new species of nodavirus which was isolated from a sample of *Culex annulirostris* mosquitoes and which we have tentatively named culannivirus (CulV). Phylogenetic analysis of the RdRp sequence placed CulV distantly from any of the currently recognized genera. By contrast, phylogenetic analysis of the capsid protein placed CulV in a clade with the currently recognized alphanodaviruses. We suggest a re-organization of the family based on the RdRp sequence.

## Materials and methods

### Ethics statement

No specific permits were required for the described field studies, however permission was sought and obtained from the traditional land owners of Bradshaw Field Training Area to conduct mosquito trapping activities in the area. These field studies did not involve endangered or protected species.

### Mosquito trapping and virus isolation

Adult mosquitoes were collected using CO_2_-baited light traps from the Bradshaw Field Training Area (BFTA) in the Northern Territory of Australia in 2014, using previously described methods [8]. Screening of mosquitoes for the presence of RNA viruses by mosquito homogenization, inoculation onto C6/36 cell monolayers and subsequent ELISA assessment was performed as previously described [9]. Successive passaging of the virus was performed by inoculating onto monolayers of C6/36 cells for 5–7 days before harvesting. Inoculations and virus passaging in C6/36 cells was performed in RPMI 1640 supplemented with 5% fetal bovine serum, 50 U/mL penicillin, 50 mg/mL streptomycin and 2 mM L-glutamine and incubation at 28 °C.

#### Sequencing and bioinformatics

RNA extraction, first and second strand cDNA synthesis, library preparation and Illumina sequencing were as previously described [10]. A virus consensus sequence of RNA1 was assembled from the data using GeneiousPro v8.1 software and Hubei noda-like virus 5 (GenBank accession number KX883080.1) as a reference sequence. The assembly was then improved with iterations of the assembly using the consensus as a reference (GenBank accession number MH794142). RNA2 was obtained using a *de novo* assembly (GenBank accession number MH794143). To construct phylogenetic trees, global multiple protein sequence alignments of RNA1 and RNA2 ORFs were performed with the Geneious Alignment feature (Blosum62 cost matrix; gap open penalty, 12; gap extension penalty, 3) [11]. A mid-point rooted phylogenetic tree was then generated using the FastTree 2.1.5 (Jones-Taylor-Thornton model) optimizing for the gamma20 likelihood and using the Shimodaira-Hasegawa test to calculate the support values [12]. Within and between mean amino acid p-distances were calculated using MEGA7 [13] from the protein alignments using with complete deletion of gaps and a shape parameter of 5.

## Results

**Fig 1:**
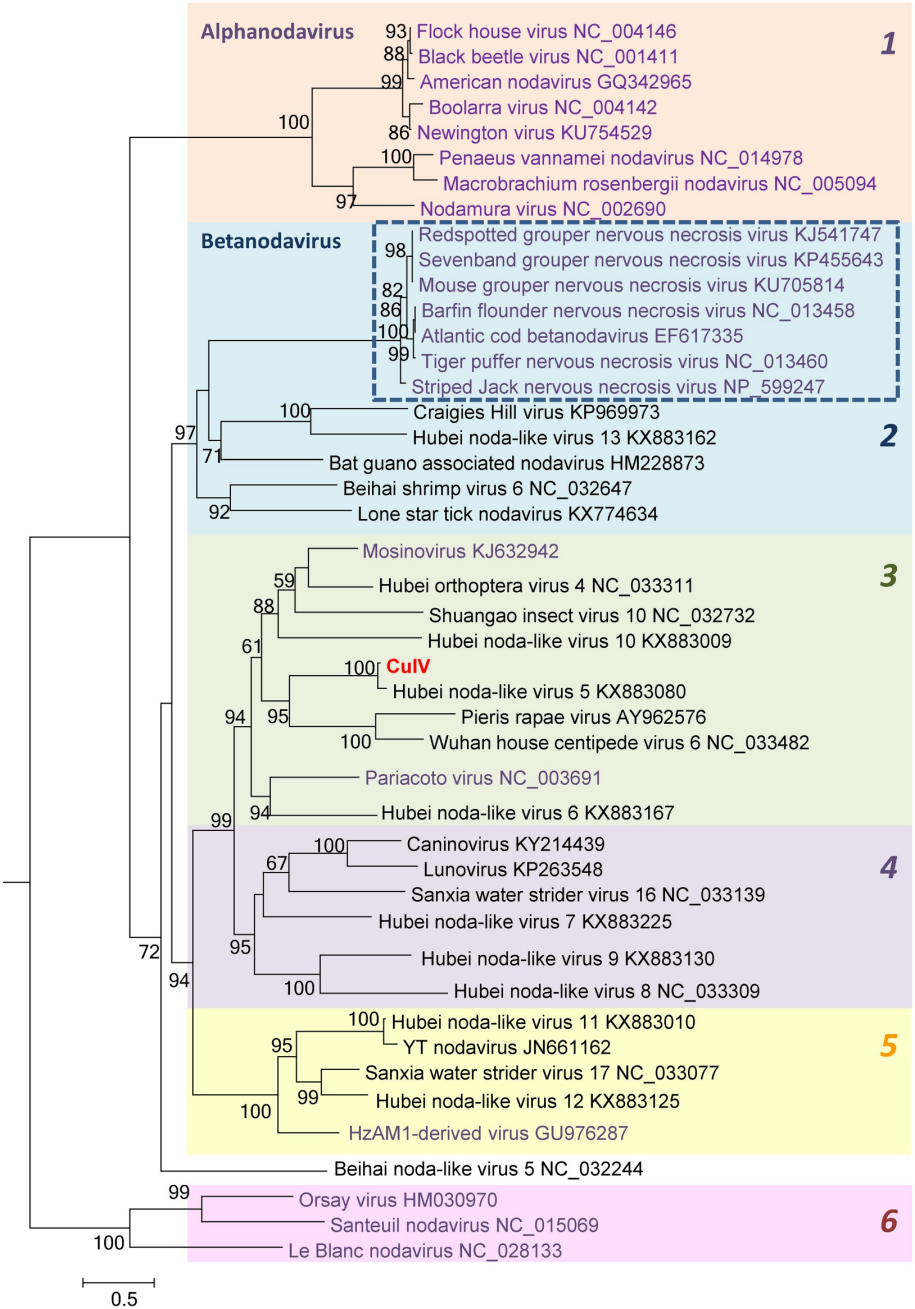
Phylogenetic analysis of the nodavirus RNA-dependent RNA polymerase sequence from RNA1. A multiple sequence alignment was performed and a tree was generated. The 6 clades are numbered and highlighted. The alphanodaviruses correspond to clade 1 and the betanodaviruses are shown in a box with a dashed outline. Biologically characterized viruses are indicated in purple and sequences from recent metagenomics studies are given in black text. Branch support values are shown.

An unidentified RNA virus was isolated during the screening of *Culex annulirostris* mosquitoes collected from the Northern Territory of Australia in 2014. We tentatively named this virus culannivirus (CulV). Preliminary analysis of deep sequencing of extracted RNA identified the virus as belonging to the *Nodaviridae* family. We performed more detailed analysis using RdRp protein sequence as it has been previously suggested that it would be preferable for taxonomic determinations [5]. Our alignments also seemed to support this, and phylogenetic analysis revealed at least 6 separate clades (Fig 1) all of which were well-supported (support values >90). The members of these clades were separated internally by genetic distances (p-distance) ranging from 0.36–0.55, whilst the groups themselves were separated by genetic distances of 0.65–0.80 (Table 1). Clade 1 corresponded to the alphanodaviruses with the notable omission of PaV. However, the grouping of PaV separate from the other alphanodaviruses has been previously observed when the RdRp was used for phylogenetic analysis [5]. This may indicate some re-assortment event in the history of this virus. Clade 2 included the betanodaviruses, but also contained additional viruses detected in a variety of arthropod hosts from recent sequencing studies [6, 14, 15]. The fish group, corresponding to the betanodavirues, composed a separate and much less diverse sub-clade (internal p-distance of 0.05). Clade 3 was a genetically diverse clade which contained PaV, MoNV and CulV in separate sub-clades. Notably, the latter two were both isolated from mosquitoes of the genus *Culex*. This clade has been greatly expanded by recent sequencing studies, with material again originating from diverse arthropod hosts [6]. Clade 4 consisted of several viruses sequenced in recent studies, but no isolate has been reported for this group. Hence, this group is one of the least biologically characterized but, interestingly, does contain two sequences identified in metagenomics studies of gut samples from a wolf and an otter [16, 17], perhaps originating from arthropods ingested by those animals. Whilst clades 3 and 4 were separate with strong support, together they also formed a larger clade (support value 99). Clade 5 includes one biologically characterized isolate obtained from the moth *Helicoverpa zea* as well as a number of nodavirus sequences from diverse insect hosts [18]. Clade 6 included sequences from virus isolates obtained from nematodes [19, 20]. These 6 clades indicate known taxonomic groupings, such as the recognized genera *Alphanodavirus* and *Betanodavirus*, or they may potentially reveal relationships based on biological characteristics which will be the basis of some future taxonomic delineation.

**Table 1:** Internal and between clade distances.

**Within group distance**			
**Taxon**		**Distance (p)**	**S.E.**
Clade 1		0.36	0.01
Clade 2		0.44*	0.01
Clade 3		0.53	0.02
Clade 4		0.55	0.02
Clade 5		0.38	0.02
Clade 6		0.52	0.02
**Between group distance**			
**Taxon 1**	**Taxon 2**	**Distance (p)**	**S.E.**
Clade 1	Clade 2	0.75	0.02
Clade 1	Clade 3	0.76	0.02
Clade 2	Clade 3	0.68	0.02
Clade 1	Clade 4	0.75	0.02
Clade 2	Clade 4	0.69	0.02
Clade 3	Clade 4	0.62	0.02
Clade 1	Clade 5	0.78	0.02
Clade 2	Clade 5	0.68	0.02
Clade 3	Clade 5	0.65	0.02
Clade 4	Clade 5	0.68	0.02
Clade 1	Clade 6	0.80	0.02
Clade 2	Clade 6	0.74	0.02
Clade 3	Clade 6	0.76	0.02
Clade 4	Clade 6	0.76	0.02
Clade 5	Clade 6	0.76	0.02

*0.05 if only betanodavirus sequences are analysed; S.E. is standard error.

**Fig 2:**
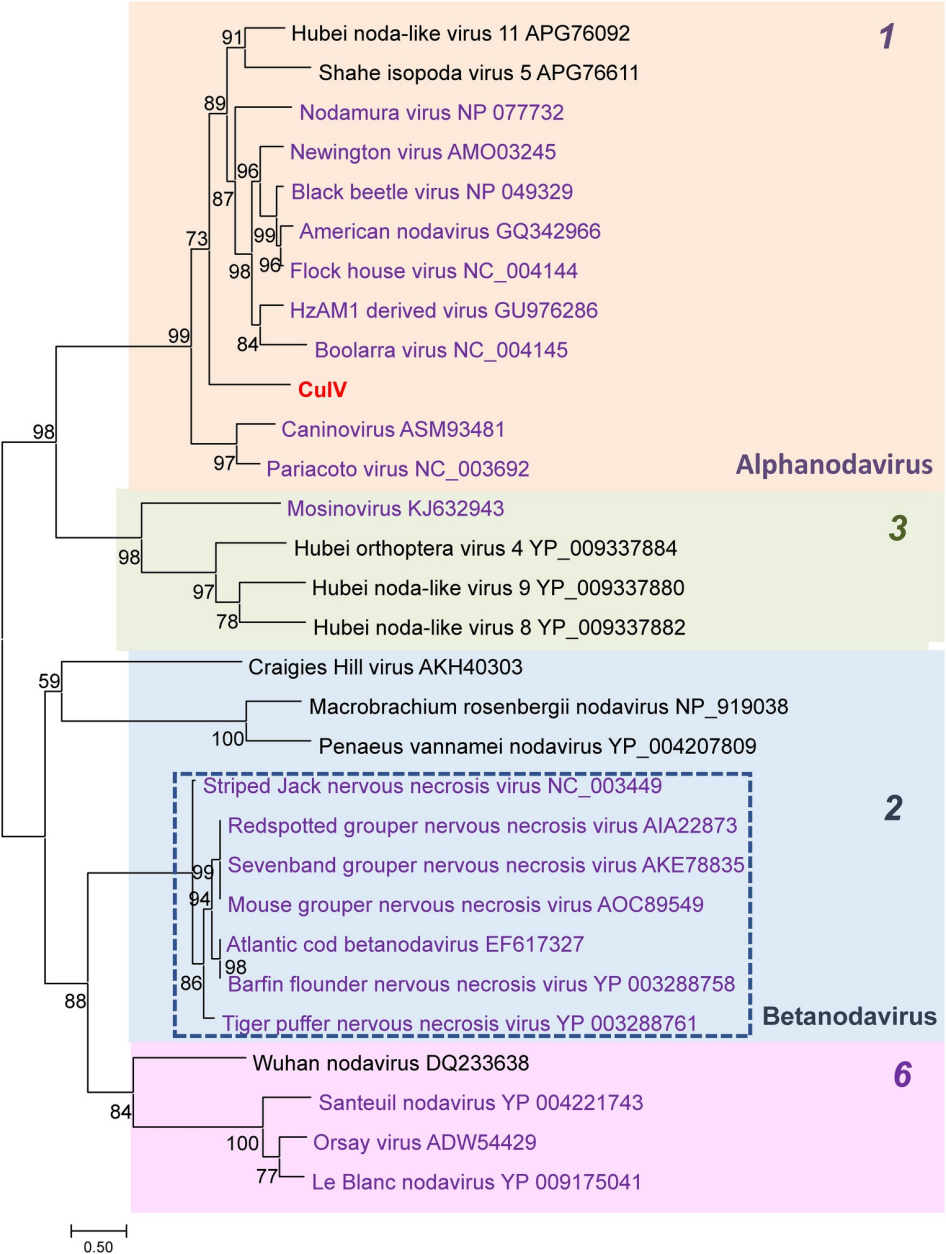
Phylogenetic analysis of the nodavirus capsid sequence from RNA2. A multiple sequence alignment was performed and a tree was generated. Viruses in the genus *Alphanodavirus* are highlighted in color. Viruses where there is some biological characterization are indicated in purple and sequences from recent metagenomics studies are given in black text. Branch support values are shown.

The capsid protein sequence has been used as the major taxonomic determinant when assigning members of the nodaviruses [21]. Analysis of the capsid structure of several nodaviruses shows that members of this virus family have diverse capsid topologies [22–27], suggesting that the capsid evolution may not be linear within the group. Available sequence was more limited for capsid. An alignment of capsid protein sequence was found to be of poor quality at the family level, making it difficult to determine the taxonomic relationships among more distantly-related nodaviruses. Phylogenetic trees constructed using this alignment revealed at least 4 clades corresponding to those identified in the RdRp tree (clades 1, 2, 3 and 6), but with weaker support values (Fig 2). Individual viruses grouped with clades that were identified for RdRp with exceptions such as CulV, which grouped with the alphanodaviruses. In addition, HzAM derived virus, *Macrobrachium rosenbergii* nodavirus and *Penaeus vannamei* nodavirus were also placed in different clades. Similar phylogenetic assignment differences with these viruses between RdRp and capsid were noted previously [5]. These data further suggest reassortment of genome RNAs during evolution.

## Discussion

The outcome of nodavirus infection is influenced by both host and virus genetic determinants. Entry of the virus into the cell involves interaction between the virus capsid and cell membrane receptors. Studies on FHV have indicated that capsid protein gamma facilitates membrane penetration during host cell entry (reviewed in [22]). Once inside the cell, the viral capsid and RdRp proteins are directly translated from the genomic RNAs using the host translation machinery. The host protein Hsp90 has been indicated to be important for efficient translation of Protein A [23]. Nodavirus replication is driven by Protein A (RdRp) which binds to the outer mitochondrial membrane and induces spherules where replication of the viral proteins takes place [24–26]. Following replication of the genomic RNAs and accumulation of the capsid proteins the virus self assembles and is released from the cell (reviewed in [27]). Many steps in the virus infection cycle involve interactions between the host and virus; hence, the genetic determinants of these interactions influence evolution of the virus thus impacting the phylogenetic resolution of the group.

The phylogenetic analysis of RdRp sequences has been reliably used for decades to determine the relationships of RNA viruses up to the family, and potentially higher, taxonomic level [28–31]. Our work also supports the use of the RdRp as being a more reliable indicator of the taxonomic relationships among the nodaviruses than capsid, as has been recently suggested [5]. The RdRp analysis placed PaV distantly from clade 1, in agreement with previous analysis, suggesting that this virus may be more accurately assigned outside this genus. It was more closely related to MoNV and CulV, both of which were isolated from mosquitoes of the genus *Culex*; the latter virus being the subject of this work. The phylogenetic analysis revealed 6 monophyletic clades, two of which were constituted either wholly or partly of the existing genera. Clade 1 corresponded to the genus *Alphanodavirus*. Clade 2 included members of the betanodaviruses and additional viruses that had been isolated from organisms other than fish. The genetic diversity of this genus is relatively small (internal p distance of 0.05) and this possibly reflects either limited sampling or a genetic bottleneck in adapting to their common fish hosts. The internal diversity of clade 2 (internal p distance of 0.44) was larger than the alphanodaviruses (internal p distance of 0.36), but smaller than the internal diversity of three of the other clades identified (i.e. clades 3, 4 and 6). Clade 2 includes viruses collected from a variety of arthropod hosts, similarly to most of the other nodaviruses, as well as fish. Virus genetic groupings generally reflect their biological characteristics such as replication strategy, genome structure, host tropism and others. The nodaviruses have a common genome structure and share the ability to infect a variety of mostly arthropod hosts, which makes it difficult to determine taxonomic groupings below the family level. Future research may reveal biological characteristics which reflect the genetic groupings identified in this study.

In consideration of the greater genetic diversity revealed by this phylogenetic analysis, which includes metagenomics and other studies, there seems to be impetus for greater taxonomic division below the family level. On the basis of a characterized virus type strain, clear monophyletic groupings, which were well-separated from other clades and had good support values, were determined. Given the level of diversity identified, future biological characterization may reveal further genera within this family *Nodaviridae*.
